# Supplementary material for: Complexome profiling of the Chlamydomonas psb28 mutant reveals TEF5 as an early PSII assembly factor
Source: Plant Cell. 2025 Mar 17;37(6):koaf055. doi: 10.1093/plcell/koaf055 (PMC12164590; doi:10.1093/plcell/koaf055)
Supplement: koaf055_Supplementary_Data [file koaf055_supplementary_data.zip › psb28_interactive.zip!/plots/intensity/qualityPlots/peptides/_Cre06.g310350.t1.2.html]

### Cre06.g310350.t1.2

Trivial name:   
SeqCoverage [median]: 3.70  
Mass: 31.04 kDa  
  
 

| Sequence | MissedCleavage | Mass [Da] | wtIntensity | muIntensity | Score |
| --- | --- | --- | --- | --- | --- |
| VADDQLGPLLR | 0 | 1195.66 | 1412.16 | 6367.20 | 125.48 |
|  |  |  | 1412.2 | 6367.2 |  |
